# LncRNA LINC01305 promotes cervical cancer progression through KHSRP and exosome-mediated transfer

**DOI:** 10.18632/aging.202565

**Published:** 2021-02-26

**Authors:** Xianxia Huang, Xuemei Liu, Bo Du, Xueling Liu, Mei Xue, Qingxin Yan, Xiaohong Wang, Qian Wang

**Affiliations:** 1 Department of Obstetrics and Gynecology, Jinan People’s Hospital Affiliated to Shandong First Medical University (Jinan City People’s Hospital), Jinan 271199, Shandong, P.R. China; 2 Department of Obstetrics and Gynecology, the Fifth People's Hospital of Jinan, Jinan 250022, Shandong, P.R. China

**Keywords:** cervical cancer, LINC01305, cancer progression, KHSRP, exosome

## Abstract

Cervical cancer (CC) is one of the deadliest female malignancies worldwide. Long non-coding RNAs (lncRNAs) are essential regulators for cancer progression. This study aimed to elucidate the role of lncRNA LINC01305 in the progression of CC. We found where LINC01305 was expressed in CC tissues and its correlation with the survival rate of CC patients. Functional experiments were performed to elucidate the effect of LINC01305 on CC. The results showed that LINC01305 was increased in CC tumor tissues and was correlated with a lower survival rate. The overexpression and knockdown of LINC01305 enhanced and inhibited the progression of CC, respectively. Additionally, the upregulation of LINC01305 promoted tumor growth in xenograft mice. Moreover, the effect of LINC01305 on CC was mediated through interacting with the RNA-binding protein, KHSRP. Furthermore, LINC01305 was mainly distributed in exosomes and was transferred to recipient cells to enhance CC progression. Lastly, LINC01305 may participate in the regulation of the stemness of CC. Taken together, the results suggest that LINC01305 promotes the progression of CC through KHSRP and that LINC01305 is released through exosomes and is involved in the stemness of CC. This study sheds light on the molecular mechanism underlying the progression of CC.

## INTRODUCTION

Cervical cancer (CC) has emerged as one of the most wide-spreading female malignancies worldwide [1]. To date, CC is the leading cause of cancer-related death for women due to its high incidence and mortality [2]. Despite advances in early diagnosis and treatment, CC remains one of the most common cancers leading to death in women, especially in developing countries [3]. Given its heterogeneity, CC is histologically categorized into three subtypes, including adenosquamous carcinoma, adenocarcinoma, and squamous cell carcinoma. At present, the front-line therapies for patients with CC include chemotherapy, radiotherapy, and surgery [1]. However, the overall survival rate of CC patients remains low [4]. Thus, it is urgent to study the molecular mechanisms underlying the carcinogenesis and progression of CC and develop new effective treatments for CC.

With a better understanding of RNAs, non-coding RNAs (ncRNAs) have drawn considerable attention from researchers. As a group of ncRNAs, long non-coding RNAs (lncRNAs), 200 nt to 100 kb in length, have been extensively studied in various physiological and pathological processes [5]. Growing evidence reveals that lncRNAs play an essential role in the progression of cancers [6]. For CC, lncRNAs exert various roles, including metastasis, apoptosis, invasion, proliferation, and epithelial-mesenchymal transition (EMT) [7]. Li et al. reported that LncRNA LINC01305 is one of the upregulated lncRNAs in CC progression [8]. Meanwhile, LINC01305 can promote EMT of CC cells through mediating the PI3K/Akt signaling pathway [9]. However, the function of LINC01305 in the progression of CC *in vitro* and *in vivo* is still not understood.

Exosomes are extracellular vesicles with diameters of 30-150 nm and can be released from almost any cell type [10]. It has been reported that tumor cells secrete at least 10-fold more exosomes than do normal healthy cells [11], and these tumor-derived exosomes can regulate the progression of cancer through transferring bioactive molecules, such as chemokines, proteins, RNAs, microRNAs (miRNAs), and lncRNAs [12]. Recently, the function of exosomal lncRNAs in cancers has been widely investigated in many cancer types, such as colorectal cancer [13], prostate cancer [14], breast cancer [15], and lung cancer [16].

Cancer stem cells (CSCs) are a small proportion of cells within the tumor with self-renewal and differentiation potential [17]. Given these essential properties, CSCs are capable of driving tumor cell proliferation and metastasis [18]. Accumulating evidence demonstrated that intercellular communication between tumor cells and CSCs are crucial for maintaining CSCs dynamic in the tumor microenvironment [19]. In addition, exosomes have been regarded as information transporters in the crosstalk between tumor cells and non-tumor cells, thereby regulating cellular processes of recipient cells [20]. However, the mechanism underlying the communication between cancer cells and CSCs has not yet been fully elucidated. Therefore, in this study, we aimed to investigate the role of lncRNA LINC01305 in the progression of CC as well as its molecular mechanism.

## RESULTS

### Biological characteristics

**Figure 1:**
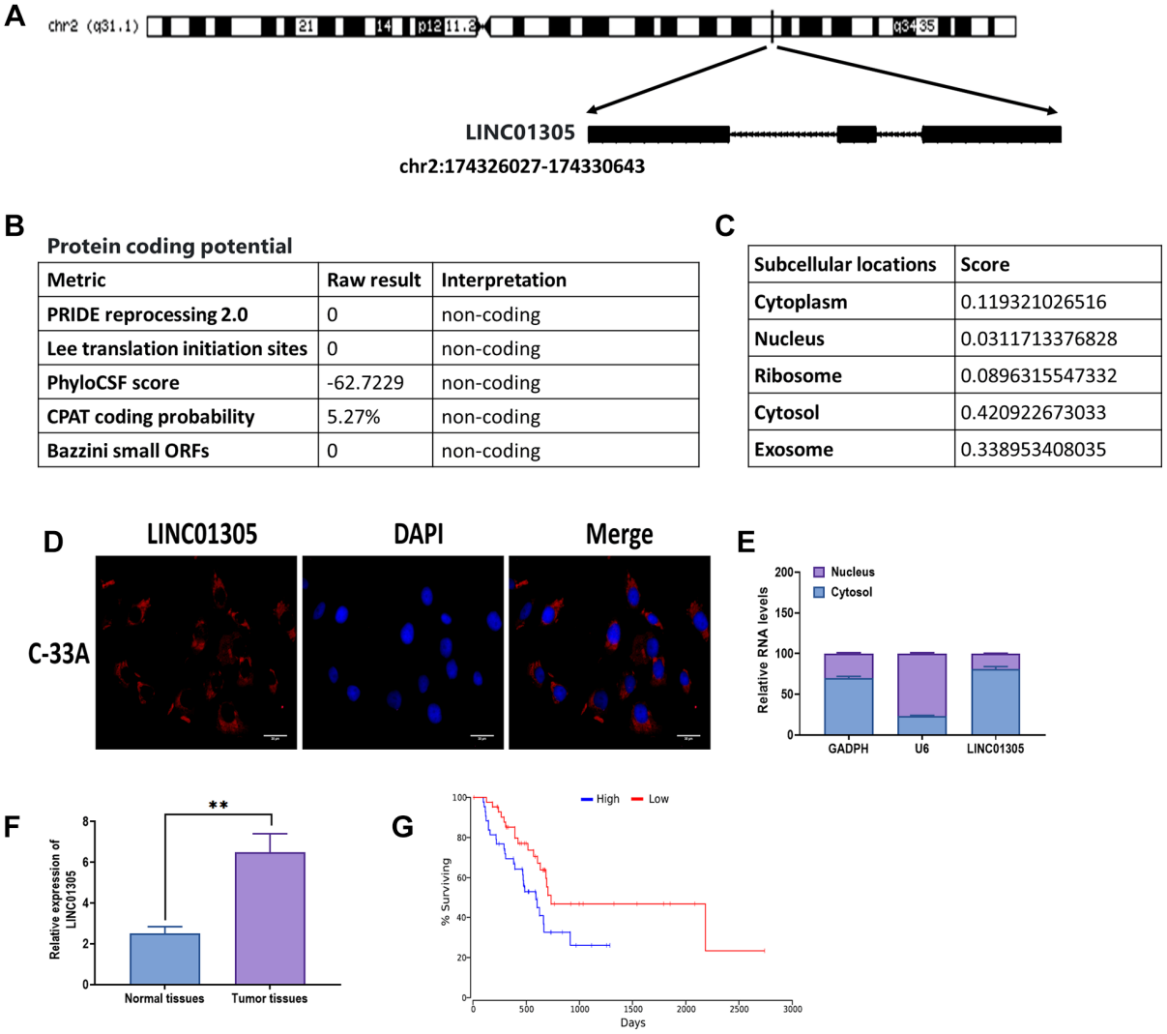
**Biological characteristics of LINC01305.** (**A**) The sequence information of LINC01305. (**B**) Prediction of protein-coding potential of LINC01305. (**C**) Prediction of subcellular location of LINC01305. (**C**) RNA fluorescence *in situ* hybridization (FISH) for the subcellular location of LINC01305. Red color represented LINC01305, and DAPI represented the nuclei. Scale bar = 30 μm. (**D**) Cell fractionation and quantitative real-time PCR for the subcellular location of LINC01305. (**E**) Expression of LINC01305 in CC tumor tissues and paired healthy tissue. (**F**, **G**) Survival rate of CC patients with high or low expression of LINC01305. * *P* < 0.05, ** *P* < 0.01, *** *P* < 0.001.

Given the potential role of LINC01305 in CC [8, 9], we aimed to investigate the role of LINC01305 in the progression of CC. First, we attempted to identify the biological characteristics of LINC01305. The analysis from the LNCipedia database [21] revealed that LINC01305 was located in the forward strand of chromosome 2 (Figure 1A). In addition, LINC01305 was 3122 bp in length and contained three exons. The protein-coding potential of LINC01305 was predicted as non-coding, according to the predictions of five metrics (Figure 1B). Also, LINC01305 was predicted to be largely expressed in the cytosol and exosome (Figure 1C). Subsequently, both RNA FISH and cell fractionation analysis showed that LINC01305 was primarily found in the cytosol of C-33A cells (Figure 1D, 1E). Next, we determined where LINC01305 was expressed in CC tissues and found that the level of LINC01305 was significantly higher in CC tissues compared with normal healthy tissues (Figure 1F). Meanwhile, the Kaplan-Meier survival analysis suggested that patients with higher expression of LINC01305 had a lower survival rate than those with a lower level of LINC01305 (Figure 1G). Therefore, these results indicated that LINC01305 may be involved in the progression of CC.

### LINC01305 promotes progression of CC *in vitro* and *in vivo*

**Figure 2:**
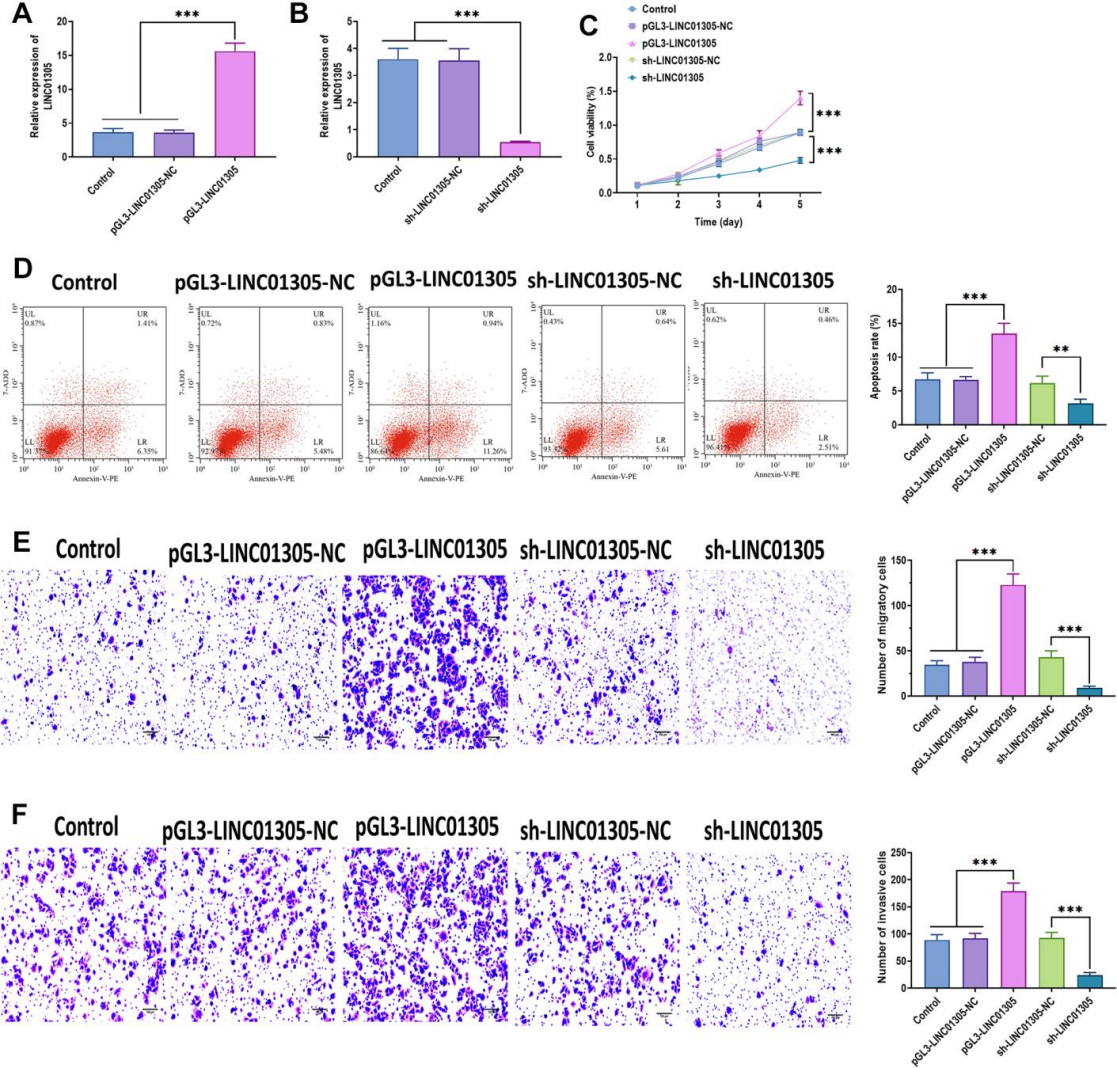
**LINC01305 promotes the progression of CC.** (**A**) Expression of LINC01305 in C-33A cells transfected with lentiviral LINC01305-expressing vectors. (**B**) Expression of LINC01305 in C-33A cells transfected with lentiviral shRNA of LINC01305. (**C**) Cell viability of C-33A cells with overexpression or silencing of LINC01305. (**D**) Apoptosis of C-33A cells with overexpression or silencing of LINC01305. (**E**) Migration of C-33A cells with overexpression or silencing of LINC01305. Scale bar = 100 μm. (**F**) Invasion of C-33A cells with overexpression or silencing of LINC01305. Scale bar = 100 μm. * *P* < 0.05, ** *P* < 0.01, *** *P* < 0.001.

**Figure 3:**
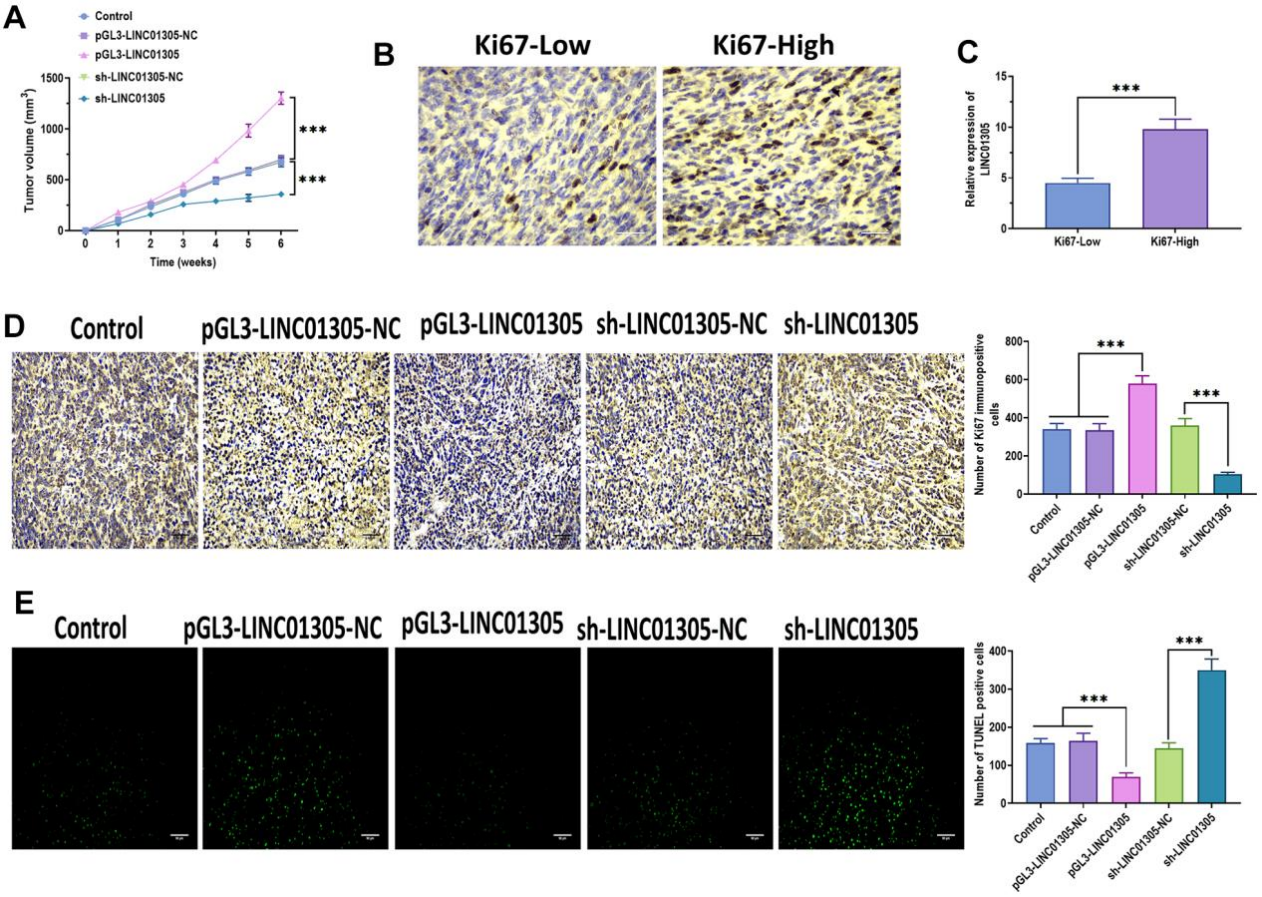
**LINC01305 promotes tumor growth *in vivo*.** (**A**) Tumor volume of xenograft mice treated with C-33A cells with overexpression or silencing of LINC01305. (**B**) Protein expression of Ki67 in CC tissues of xenograft mice. Scale bar = 30 μm. (**C**) Expression of LINC01305 in CC tissues with high or low level of Ki67. (**D**) Protein expression of Ki67 in CC tissues of xenograft mice treated with C-33A cells with overexpression or silencing of LINC01305. Scale bar = 30 μm. (**E**) Apoptosis in CC tissues of xenograft mice treated with C-33A cells with overexpression or silencing of LINC01305. Scale bar = 50 μm. * *P* < 0.05, ** *P* < 0.01, *** *P* < 0.001.

To further investigate the effect of LINC01305 on CC, we performed overexpression and knockdown of LINC01305 in C-33A cells through transfecting with lentiviral LINC01305-expressing vectors and lentiviral shRNA of LINC01305, respectively (Figure 2A, 2B). In a series of functional experiments, we found that the overexpression of LINC01305 significantly promoted cell viability, migration, and invasion, while inhibited apoptosis of C-33A cells (Figure 2C–2F). However, the silencing of LINC01305 played the opposite role (Figure 2C–2F). During the *in vivo* experiments, we observed that the tumor volume of xenograft mice injected subcutaneously with LINC01305-overexpressing C-33A cells had larger tumor tissues, whereas the silencing of LINC01305 inhibited tumor growth (Figure 3A). By immunostaining Ki67, a proliferation marker of tumor tissues [22], we observed that the expression level of LINC01305 was higher in xenograft tissues with a higher level of Ki67, while xenograft tissues with low expression of Ki67 detected a lower expression of LINC01305 (Figure 3B, 3C). Moreover, the expression of Ki67 was higher in tumor tissues of xenograft mice treated with LINC01305-overexpressing C-33A cells, while was inhibited in mice injected with LINC01305-silencing C-33A cells (Figure 3D). Furthermore, TUNEL staining revealed that lower expression of LINC01305 was associated with enhanced apoptosis in xenograft tissues (Figure 3E). Together, these results suggested that LINC01305 may promote CC progression *in vitro* and *in vivo*.

### LINC01305 interacts with KHSRP in CC

**Figure 4:**
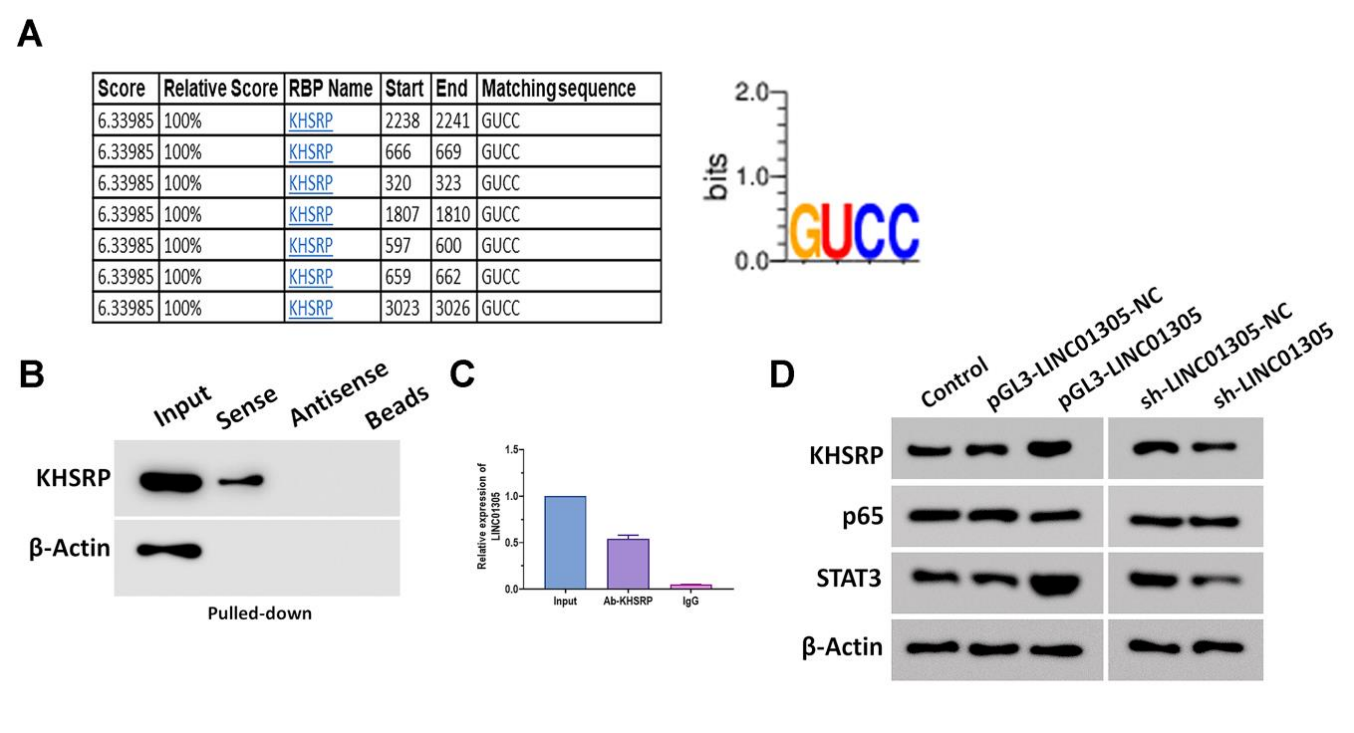
**LINC01305 interacts with KHSRP in CC.** (**A**) Prediction of RNA-binding proteins (RBPs) of LINC01305. (**B**) RNA pull-down for interaction between LINC01305 and KHSRP. (**C**) RNA immunoprecipitation assay. (**D**) Protein expressions of p65, p50, and STAT3 in C-33A cells with overexpression or silencing of LINC01305. * *P* < 0.05, ** *P* < 0.01, *** *P* < 0.001.

It has been well studied that non-coding transcripts exert an essential regulatory role in cancer by interacting with RNA-binding proteins (RBPs) in a sequence-specific pattern [23, 24]. To determine the RBPs interacting with LINC01305 in CC, we screened the potential RBPs in RBPDB [25]. Among all putative RBPs, we found that KH-Type Splicing Regulatory Protein (KHSRP) contained several conservative binding sites (GUCC) (Figure 4A), which drew our attention to explore the interaction between LINC01305 and KHSRP. Afterward, we performed RNA pull-down and RNA immunoprecipitation assays to verify this prediction, and we found that a significant enrichment of LINC01305 bound to KHSRP (Figure 4B, 4C). Growing evidence suggests that KHSRP is involved in signal transducers and activators of transcription (STAT) and NF-κB signaling pathways in various biological processes [26, 27]. Western blotting assays revealed that the protein expressions of KHSRP, p65, and STAT3 were significantly increased in C-33A cells with the overexpression of LINC01305 and decreased by the knockdown of LINC01305 (Figure 4D), suggesting that NF-κB and STAT pathways might be involved in the interaction of LINC01305 and KHSRP in CC.

### Exosomal LINC01305 promotes progression of CC

**Figure 5:**
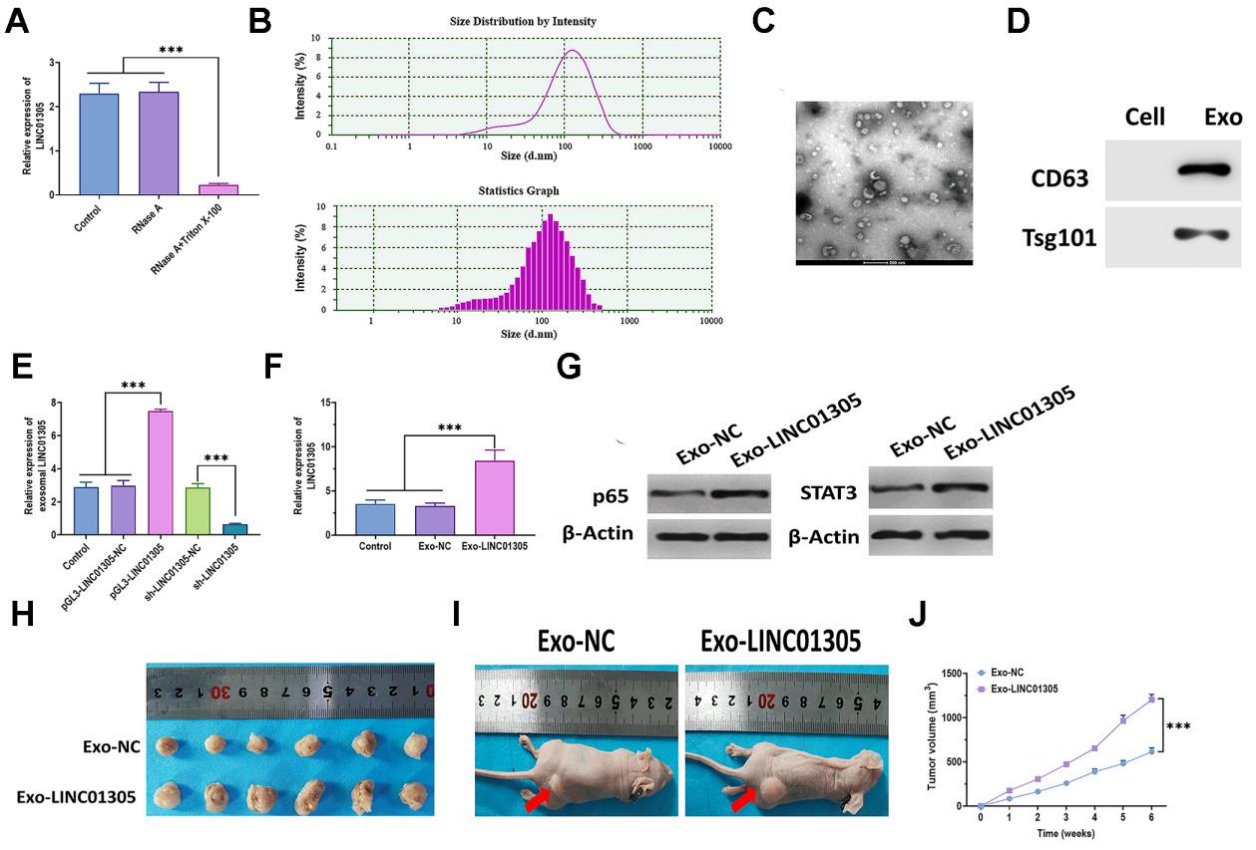
**Exosomal LINC01305 promotes the progression of CC.** (**A**) Expression of LINC01305 in C-33A cells treated with RNase A or RNase A plus Triton X-100. (**B**) Nanoparticle tracking analysis for the number and size of exosomes. (**C**) Transmission electron microscopy for the morphology of exosomes. Scale bar = 200 μm. (**D**) Protein expressions of exosomal surface markers. (**E**) Expression of LINC01305 of exosomes derived from C-33A cells with overexpression or silencing of LINC01305. (**F**) Expression of LINC01305 in C-33A cells cocultured with exosomes derived from C-33A cells with overexpression of LINC01305. (**G**) Protein expressions of p65 and STAT3 in C-33A cells cocultured with exosomes derived from C-33A cells with overexpression of LINC01305. (**H**, **I**) Representative image of tumor tissues and xenograft mice treated with exosomes derived from C-33A cells with overexpression of LINC01305. (**J**) Tumor volume of xenograft mice treated with exosomes derived from C-33A cells with overexpression of LINC01305. * *P* < 0.05, ** *P* < 0.01, *** *P* < 0.001.

As predicted, LINC01305 is mainly expressed in exosomes. Thus, we added RNase A and the combination of RNase A and Triton X-100 to the C-33A cell culture medium. The results showed that the level of LINC01305 dramatically dropped in the medium with the addition of RNase A and Triton X-100 (Figure 5A), suggesting that LINC01305 was wrapped in the membrane, rather than being released into the extracellular environment. Next, we isolated the exosomes and verified the identity of exosomes through transmission electron microscopy (TEM) and nanoparticle tracking analysis (NTA). The results revealed that the exosomes displayed a small, round shape, 84-135 nm in diameter (Figure 5B, 5C). Compared with cells, exosomes positively expressed exosomal surface markers, CD63 and Tsg101 (Figure 5D). In addition, exosomal LINC01305 was upregulated in exosomes derived from LINC01305-overexpressing cells and downregulated in exosomes derived from LINC01305-silencing cells (Figure 5E), further indicating that LINC01305 could be released from CC cells through exosomes. We found that the expression of LINC01305 was higher in C-33A cells treated with exosomes derived from LINC01305-overexpressing cells (Exo-LINC01305) than control exosomes (Exo-NC) (Figure 5F). Meanwhile, the protein expressions of p65 and STAT3 were upregulated in cells treated with Exo-LINC01305 (Figure 5G). During the *in vivo* experiments, xenograft mice injected with Exo-LINC01305 displayed larger tumor tissues and faster growth rates than those injected with Exo-NC. Collectively, these results demonstrated that LINC01305 could be released through exosomes and exosomal LINC01305 may promote the progression of CC *in vitro* and *in vivo* (Figure 5H–5J).

### LINC01305 is involved in the stemness of CC

**Figure 6:**
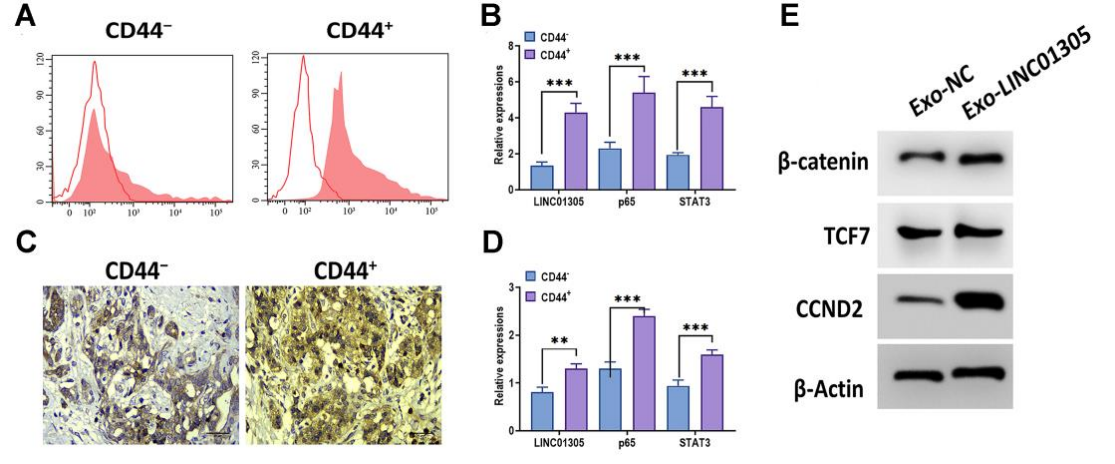
**LINC01305 is involved in the stemness of CC.** (**A**) Identification of CD44 negative and positive cells from patients with CC. (**B**) Expressions of LINC01305, p65, and STAT3 in CD44-negative or positive cells. (**C**) Expression of CD44 in CC tissues. Scale bar = 30 μm. (**D**) Expression of LINC01305, p65, and STAT3 in CD44-negative or positive tissues. (**E**) Protein expressions of β-catenin, TCF7, and CCND2 in C-33A cells cocultured with exosomes derived from C-33A cells with overexpression of LINC01305. * *P* < 0.05, ** *P* < 0.01, *** *P* < 0.001.

Since exosomal lncRNAs play a critical role in cancer stemness [13, 28, 29], we speculated that exosomal LINC01305 may be associated with the stemness of CC. By applying flow cytometry, we sorted stem-like cell populations from CC tissues through the CD44 surface marker (Figure 6A). Then, we found that the expressions of LINC01305, p65, and STAT3 were increased in CD44-positive cells, compared with CD44-negative cells (Figure 6B). Meanwhile, in CC tissues with a high level of CD44, we observed a higher level of LINC01305, p65, and STAT3, relative to CC tissues with low expression of CD44 (Figure 6C–6E). After coculturing with Exo-LINC01305, we found that the protein expressions of β-catenin, TCF7, and CCND2 were increased in C-33A cells.

## DISCUSSION

As one of the deadliest diseases, CC leads to over 300,000 deaths in women worldwide. Despite the advances in prevention, diagnosis, and treatments, the overall prognosis remains poor for patients with CC [2]. Thus, there is an urgent need to understand the molecular mechanism of the progression of CC. In the present study, we demonstrated that LINC01305 may significantly promote the progression of CC *in vitro* and *in vivo* through binding to the RNA-binding protein, KHSRP, and through the mediation of the NF-κB and STAT pathways. Also, LINC01305 was mainly distributed in exosomes and was transferred to recipient cells to enhance CC progression. Moreover, the upregulation of LINC01305 was associated with the stemness of CC through the Wnt signaling pathway.

The function of lncRNAs in cancers has been well elucidated over the past decade [30, 31]. Liang et al. reported that lncRNA DANCR functions as a competing endogenous RNA to promote CC through targeting miRNA-335-5p [32]. Also, Qing et al. demonstrated that lncRNA UCA1 suppresses invasion and proliferation of CC cells via sponging miRNA-206 [33]. In another study, Li and colleagues performed co-expression network analyses for CC-related lncRNAs and miRNAs and reported that LINC01305 is one of the top 10 up-regulated lncRNAs associated with CC progression [8]. Moreover, functional experiments suggested that LINC01305 exerts a positive role in EMT of CC cells via the PI3K/Akt signaling pathway [9]. However, the function of LINC01305 in CC progression has not been thoroughly investigated. The present study demonstrated that LINC01305 was elevated in CC tumor tissues, compared with healthy tissues, and correlated with the poor survival rate of patients with CC. As a well-studied biomarker, some lncRNAs are aberrantly expressed in tumors and have great potential for early-stage diagnosis and prognosis [34, 35]. Thus, the combinational diagnosis methods, including lncRNAs, miRNAs, sentinel lymph node (SLN) mapping for metastasis, squamous cell carcinoma antigen (SCC-Ag), and CD44, would be promising to improve diagnostic accuracy and prognosis for patients with CC [36–38]. Meanwhile, functionally, the overexpression and knockdown of LINC01305 could remarkably enhance and inhibit cell viability, migration, and invasion of CC cells, respectively. Furthermore, the pro-tumor role of LINC01305 was also observed in xenograft mice by promoting tumor growth. As such, LINC01305 may serve as a targeting biomarker for the treatment of CC.

As an important mechanism, the function of lncRNAs is mediated primarily through interacting with RBPs in a sequence-specific manner [23, 24]. Through the prediction of the database and verification via RNA pull-down and RNA RIP assays, we identified that KHSRP was a critical RBP interacting with LINC01305 to promote the progression of CC. Accumulating studies demonstrated that KHSRP plays an essential role in the regulation of cancers. For example, KHSRP can promote carcinogenesis and metastasis in non-small cell lung cancer and is associated with advanced tumor stages and shorter survival rates [39]. Also, KHSRP functions as a pro-tumor factor in pancreatic cancer [40] and colorectal cancer [41]. In addition, the downregulation of KHSRP enhances the sensitivity to 5-fluorouracil in colorectal cancer [42]. Given the multifunctional property in cancers, KHSRP and KHSRP-associated networks would be a promising target for developing the treatment of cancers, such as the decoupling interaction between KHSRP and non-coding RNAs.

Exosomes, as a critical mediator in intercellular communication, has emerged as a notable regulator in the development and progression of cancers [43]. Particularly, exosomal lncRNA has been widely studied in various cancer types [44]. As predicted, LINC01305 was expressed mainly in exosomes. In the present study, we demonstrated that LINC01305 was wrapped in exosomes derived from CC cells and that exosomal LINC01305 may increase the level of LINC01305 in recipient cells, and thereby activating the NF-κB and STAT signaling pathways. Moreover, exosomes with forced expression of LINC01305 was associated with the promotion of tumor growth in xenograft mice. These notable observations further confirm the promotive role of LINC01305 in CC and suggest that the function of LINC01305 is exerted mainly through the exosome-based mechanism.

Next, we attempted to investigate the effect of exosomal LINC01305 on recipient cells, specifically the stemness of CC. In cancer, cancer stem-like cells possess asymmetric division ability and multipotency, playing primary roles in cancer metastasis, recurrence, and chemoresistance [45]. Also, lncRNAs play an essential role in the regulation of cancer stemness [46]. In the present study, we reported that LINC01305 was upregulated in both cells and tissues with a high level of CD44, suggesting a potential role of LINC01305 in maintaining the stem-like phenotype in CC. Furthermore, Wnt signaling is an essential pathway for cancer stemness [47]. In the present study, exosomal LINC01305 activated the Wnt signaling pathway through upregulating β-catenin, TCF7, and CCND2, suggesting that Wnt signaling may be involved in the effect of LINC01305 in the stemness of CC, which may be mediated by the exosome-based intercellular communication.

In conclusion, the results demonstrated that LINC01305 promotes the progression of CC through interacting with KHSRP, is released through exosomes, and is involved in the stemness of the recipient cells. Therefore, this study provides a new understanding of the molecular mechanism underlying the progression of CC.

## MATERIALS AND METHODS

### Patient samples

One hundred and fourteen CC tissues and paired adjacent healthy tissues were collected from the patients who underwent surgery at Jinan People’s Hospital Affiliated to Shandong First Medical University between August 2014 and December 2018. Patients with CC that received treatment before surgery were excluded. The clinical characteristics of patients were summarized in Table 1.

**Table 1:** Clinical characteristics of patients with CC.

**Total number**	**114**
Age	
>60	63
≤ 60	51
HPV status	
Positive	74
Negative	40
Stage	
I-II	42
III-IV	72
Lymph node metastasis	
Positive	67
Negative	47
Histology	
Squamous cell cancer	77
Adenocarcinoma	37
Tumor size (cm)	
≤4.0	68
>4.0	46

### Cell culture

The CC cell line, C-33A, was obtained from the Cell Bank of the Chinese Academy of Sciences (Shanghai, China). The C-33A cells were cultured in Roswell Park Memorial Institut-1640 (RPMI-1640) medium, supplemented with 10% fetal bovine serum (FBS), 100 U/mL penicillin, and 100 μg/mL streptomycin (Sigma-Aldrich, USA). Cells were placed in an incubator with 5% CO_2_ at 37° C.

### Quantitative real-time PCR

Total RNA of tissues, cells and exosomes was isolated using TRIzol™ Reagent (Thermo Fisher Scientific, USA). Next, first-stand cDNA was synthesized using SuperScript III First-Strand Synthesis SuperMix (Thermo Fisher Scientific, USA) according to the manufacturer’s instructions. RNA integrity and concentration were measured using the anoDrop™ 2000 (Thermo Scientific, USA). PCR reactions were carried out using the SYBR Premix Ex Taq Reagent Kit (Takara, Japan) on a 7900 Real-Time PCR Platform (Applied Biosystems, USA). Primers were used as follows: LINC01305: (F) 5’-CCACGCAGCTCTCCAACACTC-3’, (R) 5’-TTTGGGCGACTACAGAATCCA-3’; β-actin: (F) 5’- CACCATTGGCAATGAGCGGTTC-3’, (R) 5’-AGGTCTTTGCGGATGTCCACGT-3’. β-Actin was used as the reference gene and 2^−ΔΔCt^ method was used to analyze the relative expressions.

### Subcellular localization

Cell fractionation was carried out using the PARIS™ Kit (Thermo Fisher Scientific, USA) according to the manufacturer’s instructions. The expression of LINC01305 was determined as mentioned above.

### Fluorescence *in situ* hybridization (FISH)

The FISH assay was performed using the FISH Tag™ RNA Multicolor Kit (Thermo Fisher Scientific, USA) according to the manufacturer’s instructions. Images were taken using SP8 laser confocal microscopy (Leica, Germany).

### Western blotting and immunohistochemistry

Total protein of cells and exosomes was isolated using SDS Lysis Buffer (Sigma-Aldrich, China). Western blotting assay was performed as previously described [48]. The first antibodies were obtained from Abcam (Abcam, USA) as follows: KHSRP (1:500), β-actin (1:1000), p65 (1:500), STAT3, CD63(1:500), Tsg101 (1:1000), β-catenin (1:1000), TCF7 (1:500), and CCND2 (1:500). The protein expression of β-actin was used to calculate the relative protein expression. Immunohistochemistry assay was performed as previously described [49, 50]. Antibodies against CD44 and Ki67 were used to detect the protein expression in tissues of xenograft mice at day 16 after receiving treatments as indicated.

### Flow cytometry

Cells subjected to treatments as indicated were reaped after 48 hours. Cell apoptosis was determined using flow cytometric analysis with the Annexin V-FITC Apoptosis Detection Kit (Sigma-Aldrich, USA) according to the manufacturer’s instructions. Flow cytometry assay was performed on the CytoFLEX Flow Cytometer (Beckman Coulter, USA). All experiments included at least three replicates.

### Cell transfection

LINC01305 cDNA was synthesized and cloned into lentiviral pGL3-Basic vectors by GenePharma (Shanghai, China). Lentiviral shRNA of LINC01305 was obtained from GenePharma (Shanghai, China). Cell transfection was carried out using the Lipofectamine™ 3000 Transfection Reagent (Invitrogen, USA) according to the manufacturer’s instructions.

### TUNEL staining assay

The TUNEL staining assay was performed using the Apo-Direct TUNEL Assay Kit (Abcam, USA) according to the manufacturer’s instructions. Tissues of xenograft mice were collected on day 16 after receiving treatments as indicated.

### Cell function experiments

Cell viability was investigated using the Cell Counting Kit 8 (WST-8 / CCK8) (Abcam, USA) according to the manufacturer’s instructions. Cell invasion and migration abilities were determined using the CytoSelect™ 24-Well Cell Migration and Invasion Assay kit (Cell Biolabs Inc, USA) according to the manufacturer’s instructions. Invasive or migratory cells were fixed in methanol and stained with crystal violet (Abcam, USA).

### RNA pull-down and RNA RIP

RNA pull-down assay was performed using the Pierce™ Magnetic RNA-Protein Pull-Down Kit (Thermo Fisher Scientific, USA) according to the manufacturer’s instructions. Antibodies against KHSRP and β-actin (Abcam, USA) were used. RNA immunoprecipitation assay was performed using the Magna RIP™ RNA-Binding Protein Immunoprecipitation Kit RNA Immunoprecipitation (RIP) Kit (Sigma-Aldrich, USA) according to the manufacturer’s instructions. Antibodies against KHSRP and IgG were obtained from Abcam (Abcam, USA).

### Exosome experiments

Exosomes were isolated from the cell culture medium using Total Exosome Isolation Reagent (Thermo Fisher Scientific, USA) according to the manufacturer’s instructions. The morphology of exosomes was determined using transmission electron microscopy (Thermo Scientific, USA), as previously described [51]. The number and size of exosomes were determined using the NanoSight NS300 instrument (Malvern Instruments Ltd. UK). Exosomal surface markers, CD63 and Tsg101, were determined by western blotting as mentioned above. During the *in vitro* experiments, exosomes (2μg) were used to coculture with cells.

### Xenograft mouse model

C57BL/6 athymic nude mice (6-8 weeks old) were obtained from Laboratory Animal Centre of Sun Yat-sen University (Guangzhou, China) and housed in the standard conditions. C-33A (1 × 10^6^) cells or exosomes (5 μg) were subjected to treatments as indicated and the treatments were injected into the flank subcutaneously, and tumor volumes were monitored weekly. Tumor volume was quantified using the formula: V = (L × W^2^) × 0.5. After 6 weeks, mice were sacrificed.

### Statistical analysis

Data were represented as mean ± SEM. Survival rate of patients with different levels of LINC01305 were calculated using the Kaplan-Meier estimate with log-rank test. Comparison between two groups was performed using the Student’s t-test or one-way ANOVA. *P* < 0.05 was considered statistically significant.

### Ethical statements

Written informed consent was obtained from all patients with CC. The study was approved by the Ethical Committee of Jinan People’s Hospital Affiliated to Shandong First Medical University. All animal studies were performed with the approval of the Institutional Animal Care and Use Committee of Jinan People’s Hospital Affiliated to Shandong First Medical University.

### Data availability statement

The original contributions presented in the study are included in the article/supplementary material, further inquiries can be directed to the corresponding author/s.
